# Old-new ERP effects and remote memories: the late parietal effect is absent as recollection fails whereas the early mid-frontal effect persists as familiarity is retained

**DOI:** 10.3389/fnhum.2015.00532

**Published:** 2015-10-14

**Authors:** Dimitris Tsivilis, Kevin Allan, Jenna Roberts, Nicola Williams, John Joseph Downes, Wael El-Deredy

**Affiliations:** ^1^School of Psychology, University of LiverpoolLiverpool, UK; ^2^School of Psychology, University of AberdeenAberdeen, UK; ^3^School of Psychological Sciences, University of ManchesterManchester, UK; ^4^School of Biomedical Engineering, University of ValparaisoValparaiso, Chile

**Keywords:** recognition memory, event-related potentials, FN400, LPE, episodic memory

## Abstract

Understanding the electrophysiological correlates of recognition memory processes has been a focus of research in recent years. This study investigated the effects of retention interval on recognition memory by comparing memory for objects encoded four weeks (remote) or 5 min (recent) before testing. In Experiment 1, event related potentials (ERPs) were acquired while participants performed a yes-no recognition memory task involving remote, recent and novel objects. Relative to correctly rejected new items, remote and recent hits showed an attenuated frontal negativity from 300–500 ms post-stimulus. This effect, also known as the FN400, has been previously associated with familiarity memory. Recent and remote recognition ERPs did not differ from each other at this time-window. By contrast, recent but not remote recognition showed increased parietal positivity from around 500 ms post-stimulus. This late parietal effect (LPE), which is considered a correlate of recollection-related processes, also discriminated between recent and remote memories. A second, behavioral experiment confirmed that remote memories unlike recent memories were based almost exclusively on familiarity. These findings support the idea that the FN400 and LPE are indices of familiarity and recollection memory, respectively and show that remote and recent memories are functionally and anatomically distinct.

## Introduction

Event-related potentials (ERPs) have been a valuable tool in the investigation of the neural correlates of recognition memory by identifying patterns of activity that discriminate between successfully classified old and new items, the so called old/new effects. A significant number of these studies have also examined the electrophysiological correlates of recollection and familiarity (Smith, 1993; Allan and Rugg, 1997; Düzel et al., 1997; Rugg et al., 1998; Curran and Cleary, 2003; for a review, see Rugg and Curran, 2007), the two memory processes, which, according to some theoretical accounts, underpin recognition judgements. Despite their differences, dual-process models propose that one of the processes, recollection, involves the retrieval of contextual details associated with a specific item whereas the other, familiarity, is closely related to memory strength and reflects the subjective feeling than an item has been previously experienced, even when specific details of the initial learning episode cannot be retrieved (Mandler, 1980; Tulving, 1985; Jacoby, 1991; Hintzman and Curran, 1994; Yonelinas, 2002; Diana et al., 2007; Eichenbaum et al., 2007; Montaldi and Mayes, 2010; Ranganath, 2010) Although dual process theories have not gone unchallenged (e.g., Donaldson, 1996; Squire et al., 2007), behavioral, neuropsychological and neuroimaging evidence suggest that recollection and familiarity can be dissociated on the basis of their differential sensitivity to experimental manipulations and reliance on distinct anatomical and functional neuronal networks (Henson et al., 1999; Düzel et al., 2001; Mayes et al., 2004; Aggleton et al., 2005; Yonelinas et al., 2005; Montaldi et al., 2006; Bowles et al., 2007; Tsivilis et al., 2008; Vann et al., 2009; Aly et al., 2011; Addante et al., 2012a).

ERP studies have provided strong support for dual process theories. Recollection has been associated with a parietal old/new effect (often left-sided), which onsets at around 400–500 ms following stimulus presentation. In the Remember/Know paradigm (Tulving, 1985; Gardiner and Java, 1991), Remember responses, which are thought to reflect recollection, elicit the late parietal effect (LPE) in contrast to familiarity-based Know responses (e.g., Smith, 1993; Düzel et al., 1997; Curran, 2004; Yu and Rugg, 2010). Similarly, LPE amplitude is greater when recognition is accompanied by the retrieval of source information and multiple and more precise episodic details (Wilding and Rugg, 1996; Wilding, 2000; Vilberg et al., 2006; Murray et al., 2015). By contrast, familiarity has been linked to a frontally distributed effect, which appears from 300–500 ms after stimulus onset and is known as the early mid-frontal effect or FN400 (e.g., Curran, 2000). The effect is elicited by Know responses and is sensitive to the similarity between test cue and the original studied item and to manipulations of memory strength such as subjective feelings of confidence (Curran, 2004; Azimian-Faridani and Wilding, 2006; Groh-Bordin et al., 2006; Woodruff et al., 2006; Yu and Rugg, 2010; Addante et al., 2012b). The functional dissociation between the LPE and the FN400 is further highlighted in electrophysiological studies involving memory impaired patients with damage restricted to the hippocampus, a structure known to affect recollection but not familiarity (e.g., Aggleton and Brown, 1999; Diana et al., 2007; Montaldi and Mayes, 2010). In these patients the FN400 is still present whereas the LPE is absent (Düzel et al., 2001; Addante et al., 2012a).

Although there is an overall consensus regarding the link between recollection and the LPE, the processes reflected in the FN400 have been the subject of intense debate. An alternative interpretation considers the FN400 an electrophysiological correlate of conceptual priming based on findings that link the effect with the existence of semantic representations (e.g., Paller et al., 2007; Voss and Paller, 2007; see Voss et al., 2012 for a recent discussion) However, this account cannot readily explain the absence of an FN400 for studied items which although meaningful are not recognized at test (Rugg et al., 1998; Tsivilis et al., 2001; Yu and Rugg, 2010) as well as evidence that the FN400 is modulated by perceptual changes between study and test (e.g., Schloerscheidt and Rugg, 2004; Groh-Bordin et al., 2006) but not by factors that affect conceptual priming (Stenberg et al., 2009). The topography of conceptual priming ERP effects and the FN400 are also different suggesting the involvement of at least partially distinct neural generators (Bridger et al., 2012).

Existing knowledge is limited, however, by the fact that the vast majority of ERP studies have examined retrieval at very short retention delays-usually within minutes after study. Therefore, it is unclear whether the electrophysiological correlates of recognition memory change as the delay between study and test increases and whether, by extension, the neural networks that support the retrieval of recent and remote memories differ. The latter has been an issue of intense debate. Memories are believed to undergo consolidation, a term broadly used to describe both short and long-term processes aimed at stabilizing the mnemonic representation (e.g., Squire and Alvarez, 1995; Wixted, 2004). Short-term, trace strengthening processes operate at the cellular level and are complete within minutes or hours after learning whereas long-term processes involved in long-term (or systems) consolidation are presumed to last for months, years or even decades (Dudai, 2004). According to what is sometimes referred to as the standard model (Squire and Alvarez, 1995), systems consolidation involves the gradual, time-dependent reorganization of the memory trace from one that initially requires the participation of the hippocampus to one that depends solely on direct connections between cortical storage sites. An alternative account, suggests that only semantic, factual memories undergo long-term consolidation whereas remote episodic memories require hippocampal involvement, which ensures that they retain their episodic character in that they are accompanied by contextual details and a sense of vividness (Nadel and Moscovitch, 1997; Nadel et al., 2012).

Behavioral differences between remote and recent memories are evident within relatively short retention intervals. Prospective memory studies have shown that recollection tends to decline faster than familiarity response rates at least within the first weeks after encoding (Gardiner and Java, 1991) reflecting the fact that item memory is retained for longer than memory for contextual features (Dudukovic and Knowlton, 2006).

There have only been a handful of studies to examine recognition ERPs at longer retention intervals (Curran and Friedman, 2004; Wolk et al., 2006; Jaeger et al., 2009). Both Curran and Friedman (2004) and Wolk et al. (2006), who looked at immediate and one day old memories failed to find any delay-related modulation of the FN400 and LPE. By contrast, Roberts et al. (2013) found an attenuation of LPE amplitude for week-old recollected memories compared to immediate recollection (see also Jaeger et al., 2009). A follow up, behavioral experiment revealed that the reduced parietal activity likely reflected the loss of episodic details in remote recollection, an explanation further supported by findings of a positive correlation between amount recollected and LPE magnitude in immediate testing (Wilding, 2000; Vilberg et al., 2006). In agreement with the earlier studies (Curran and Friedman, 2004; Wolk et al., 2006; Roberts et al., 2013) found no evidence that the FN400 was affected by delay.

The aim of the present study was to explore further the effects of retention interval on the electrophysiological correlates of recognition memory by comparing ERPs to recent memories, formed minutes prior to retrieval, to those elicited by remote memories acquired four weeks before testing. Importantly, we employed a longer interval for remote memory testing than the previous electrophysiological studies, in an attempt to increase the likelihood of identifying time-related changes beyond those previously reported. Testing memory four weeks after encoding likely represents the longest delay at which remote recognition ERPs can be obtained in prospective memory paradigms under experimental conditions that do not involve encoding manipulations aimed to boost remote recognition performance. Adopting identical encoding conditions for recent and remote items avoids possible confounds related to initial learning differences and allows direct comparisons to be made between the present study and the greater ERP recognition memory literature.

It should also be noted, that the present ERP study did not require participants to distinguish between recollection and familiarity based, remote and recent memories. Because recent memories tend to be overwhelmingly supported by recollection and remote memories are more likely to be familiarity based (e.g., Gardiner and Java, 1991; Roberts et al., 2013), it would not have been possible to acquire sufficient number of trials to obtain reliable ERPs for all the different classes of memory responses [see also Suchan et al. (2008) and Roberts et al. (2013), for similar concerns]. Interpretation of the ERP data therefore relied instead upon existing knowledge of the functional significance of the FN400 and the LPE, supplemented by the findings of a second, behavioral-only experiment, which allowed an exact measurement of recollection and familiarity scores at the two delays.

## Experiment 1

### Method

#### Participants

Nineteen volunteers took part in this experiment in exchange for course credits or payment of £5 per hour. All were right-handed with normal or corrected-to-normal vision. Participants provided informed consent for the study which was approved by the University of Liverpool, School of Psychology Ethics Committee. Data from three participants were excluded from all subsequent analyses: One participant was rejected for failing to follow instructions and two for having fewer than 16 artifact-free trials in at least one of the experimental conditions (see ERP methods). Data are reported from the remaining 16 participants (12 female; age range = 18–36 years; mean age = 20 years).

#### Stimuli

The stimuli comprised 450 colored clip art pictures of animate and inanimate objects. Of those, 260 were taken from Rossion and Pourtois (2004)^1^ whereas the remaining were downloaded from various internet sites. Pictures were randomly divided into three sets of 150 items. Two of these sets were presented at both study and test (“old”) and one only at test (“new”). Of the two old sets, one was presented at the first session (remote items) and the other at the second session (recent items). The use of the three sets as old/new, remote/recent was counterbalanced across participants.

#### Procedure

The experiment consisted of two sessions. During the first session, participants studied one set of picture items. In the second session they studied a second set and following that they completed a recognition memory test, which included remote, recent and new items. The average period between the remote study session and test was 28 days (*SD* = 0.91; minimum interval: 26 days, maximum interval: 30 days) whereas for items studied in the second session the average delay between the end of study and the beginning of the test phase was 5 min (*SD* = 3.69; minimum delay: 2 min, maximum delay: 16 min).

The structure of both encoding and test trials was the same. A white square was continuously displayed at the center of the computer monitor for the duration of the experiment. The horizontal and vertical visual angles of the square were approximately 4° at 80 cm viewing distance. Each trial commenced with the presentation of an “O” character at the center of the screen for 300 ms which acted as a warning signal that a stimulus was imminent. The character disappeared 150 ms before the stimulus picture. Each object picture was shown centrally within the white square background. A small, central fixation cross was superimposed on the image and remained visible for an additional 1400 ms after the stimulus had disappeared. The screen was then blanked for 1550 ms until the end of the trial. Participants were asked to retain fixation while the cross was on display. The encoding task was the same at both study phases and required participants to indicate for each picture whether it felt “pleasant” or “not so pleasant”, by pressing either the A or L keys on the keyboard. At test, participants used the same keys to indicate whether an item had been seen before during the experiment (irrespective of whether it was recent or remote) or whether it was new. The assignment of keys to responses was counterbalanced across participants.

#### ERP Recording and Analysis

EEG was recorded continuously during both study and test phases from 31 silver/silver-chloride electrodes. Twenty nine of these electrodes were embedded in an elastic cap (Easy cap:^2^) and two were placed on the left and right mastoid bones. The locations of the cap electrodes were based on the International 10–20 system and included three midline sites (Fz, Cz and Pz), as well as 26 homotopic sites over the two hemispheres (left/right: Fp1/Fp2, F1/F2, F3/F4, F5/F6, F7/F8, C3/C4, C5/C6, T7/T8, CP5/CP6, P3/P4, P5/P6, P7/P8, O1/O2). Electroculogram (EOG) was recorded from two electrodes placed above and below the right eye (vertical EOG) and two on the outer canthi (horizontal EOG). All channels were amplified with a bandpass of 0.05–30 Hz and impedances were kept below 5kΩ. Data were acquired at a rate of 4 ms per point, using Fz as online reference. Offline, data were re-referenced to linked mastoids and Fz recordings were algebraically restored. Waveforms were segmented into 2000 ms epochs from stimulus onset with a 500 ms pre-stimulus period serving as baseline.

## Results and Discussion

### Behavioral Results

Hit and false alarm scores are presented in Table 1. As expected, participants remembered fewer remote than recent pictures (*t*_(15)_ = −9.63, *p* < 0.001.) Remote hit rates were greater than false alarms (*t*_(15)_ = 6.82, *p* < 0.001) indicating above chance performance after four weeks. Reaction times (RTs) for recent hits (*M* = 813.2, *SD* = 232.3), remote hits (*M* = 965.9, *SD* = 338.0) and correct rejections (*M* = 935.6, *SD* = 297.3) were analyzed in a repeated measures ANOVA and were significantly different (*F*_(1.56,23.42)_ = 15.50, *p* < 0.001). Pairwise comparisons revealed faster responses to recent compared to remote hits and correct rejections (both at *p* < 0.01). RTs to remote hits and correct rejections did not differ from each other (*p* = 0.267).

**Table 1:** **Behavioral results for Experiments 1 and 2**.

	**Experiment 1 (*N* = 16)**		
	Recent hits	Remote hits	False alarms
Recognition	0.82	0.42	0.29
	(0.12)	(0.13)	(0.16)
	**Experiment 2 (*N* = 14)**		
	Recent hits	Remote hits	False alarms
Recognition	0.84	0.30	0.17
	(0.10)	(0.17)	(0.15)
Recollection	0.55	0.06	0.02
	(0.21)	(0.10)	(0.04)
Familiar	0.29	0.24	0.15
	(0.19)	(0.17)	(0.15)

*Mean recent and remote hit and false alarm rates in Experiments 1 and 2. Standard deviations are shown in parentheses*.

### ERP Analysis

Electrophysiological analyses were performed on data from twelve electrode sites which, based on previous studies (e.g., Rugg et al., 1998), capture the effects of interest. These involved frontal and parietal, left and right hemisphere sites (F7, F5, F3, F4, F6, F8, P7, P5, P3, P4, P6, and P8). Consistent with the aim of the study, two time windows were selected on the basis of previous evidence: the 300–500 ms period for the FN400 and 600–800 ms for the LPE. The analyses first involved an overall repeated measures ANOVA with the following factors: condition (remote hits vs. recent hits vs. correct rejections), region (frontal vs. parietal), hemisphere (left vs. right) and site (superior vs. middle vs. inferior). Significant main effects of condition or interactions involving the factor of cognition were investigated further in follow-up repeated measures ANOVAs. Degrees of freedom in ANOVAs violating the assumption of non-sphericity were corrected using the Greenhouse-Geisser procedure. Only ERP results involving the factor of condition are reported.

### ERP Results

**Figure 1:**
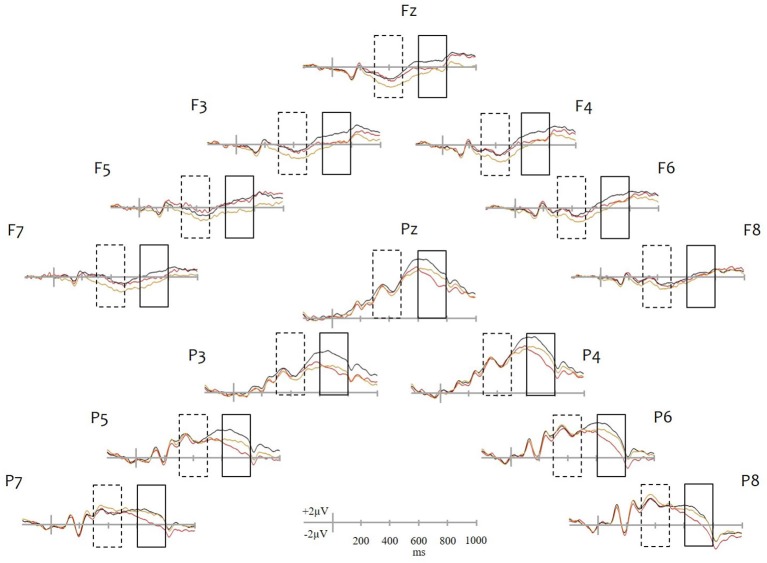
**Grand average waveforms**. ERP plots are shown for selected frontal (Fz, F3, F4, F5, F6, F7, F8) and parietal (Pz, P3, P4, P5, P6, P7, P8) sites. Recent hits are drawn in black, remote hits in red and correct rejections in yellow. Dashed and solid line rectangles identify the 300–500 ms and 600–800 ms periods, respectively, in which ERPs were analyzed. Note: With the exception of the two midline sites (Fz and Pz), all sites shown were included in the analyses.

Average waveforms can be seen in Figure 1. Recent hits elicited more positive going activity relative to correct rejections at frontal electrodes from approximately 250 ms following stimulus onset. Amplitude differences at parietal sites emerged later at around 500 ms and were greater and longer lasting at left parietal sites. Remote old-new effects were distinct from recent old-new effects. Remote hits were more positive going than correct rejections only between 300–500 ms at frontal sites. Differences between recent and remote hits appeared at around 500 ms, were restricted to parietal sites and were characterized by greater positivity for recent than remote memories.

### FN400: 300–500

The overall ANOVA revealed significant differences between conditions in this time window (main effect of condition: *F*_(2,30)_ = 3.92, *p* = 0.031) and was followed by pairwise contrasts. The comparison between recent hits and correct rejections revealed significant effects of condition (*F*_(1,15)_ = 11.35, *p* = 0.004) and a condition × region interaction (*F*_(1,15)_ = 12.46, *p* = 0.003). At this time period, significant differences between recent hits and correct rejections were present at frontal (*F*_(1,15)_ = 20.74, *p* < 0.001) but not parietal sites (*F*_(1,15)_ = 0.011, *p* = 0.917). The condition × site (*F*_(2,30)_ = 7.68, *p* = 0.002) and the three-way condition × region × site interaction (*F*_(2,30)_ = 7.42, *p* = 0.002) were also significant, mainly due to differences being larger at superior (closer to the midline) frontal sites.

Despite a strong trend, the comparison between remote hits and correct rejections did not reveal a significant main effect (*F*_(1,15)_ = 3.83, *p* = 0.069). However, the condition × region interaction was significant (*F*_(1,15)_ = 7.17, *p* = 0.017) as a result of significant differences at frontal (*F*_(1,15)_ = 8.97, *p* = 0.009) but not parietal (*F*_(1,15)_ = 0.54, *p* = 0.475) sites. The condition × site interaction (*F*_(2,30)_ = 5.66, *p* = 0.008) was also significant, with greater differences at superior sites. The direct comparison between recent and remote hits did not reveal any differences.

### LPE: 600–800

The main effect of condition (*F*_(2,30)_ = 16.85, *p* < 0.001) was significant. Subsequently, the comparison of recent hits to correct rejections also revealed a main effect of condition (*F*_(1,15)_ = 17.62, *p* < 0.001) with recent hits showing greater positivity than correct rejections. The condition × site and condition × region × hemisphere × site interactions were also significant (*F*_(1.28,19.23)_ = 5.52, *p* = 0.023 and *F*_(2,30)_ = 6.59, *p* = 0.004, respectively), which largely reflected the fact that differences at left inferior frontal sites tended to be greater than those at right inferior parietal sites.

The comparison between remote hits and correct rejections did not reveal a main effect of condition (*F*_(1,15)_ = 1.38, *p* = 0.26), but both the condition × region (*F*_(1,15)_ = 20.83, *p* < 0.001) and the three-way, condition × region × site interactions (*F*_(2,30)_ = 4.30, *p* = 0.023) were significant. The follow up ANOVA showed condition differences at parietal sites only (*F*_(1,15)_ = 16.25, *p* = 0.001), with remote hits being more negative going than correct rejections. This posterior effect was more pronounced at middle and inferior sites as suggested by the significant condition × ite interaction (*F*_(2,30)_ = 5.71, *p* = 0.008).

Finally, the direct comparison between remote and recent hits found a main effect of condition (*F*_(1,15)_ = 26.77, *p* < 0.001) as recent hits were more positive going than remote hits. The condition × region interaction was also significant (*F*_(1,15)_ = 13.12, *p* = 0.003) with amplitude differences greater at parietal sites.

### Topographical Analyses

**Figure 2:**
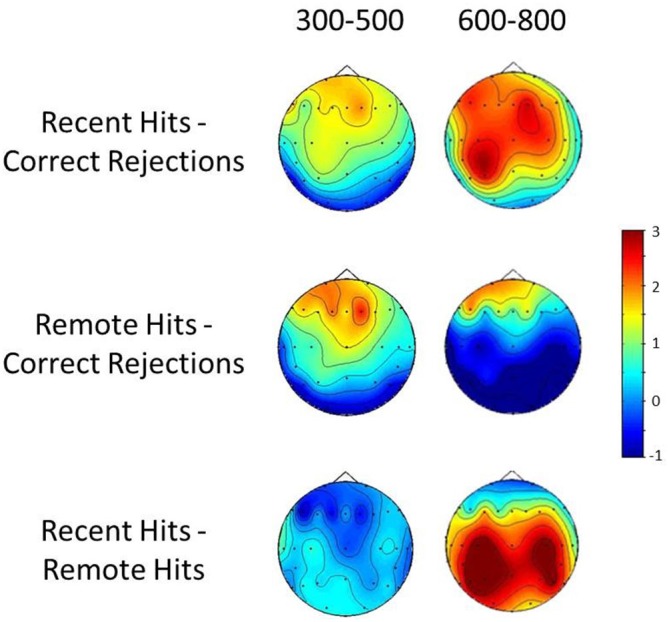
**Topographic distribution difference maps for the 300–500 ms and 600–800 ms periods**. Top row: Recent hits-Correct rejections; Middle-row: Remote hits-Correct rejections; Bottom row: Recent hits-Remote hits.

A repeated measures ANOVA was also performed to compare the scalp distributions of recent and remote old-new differences. Topographical distribution difference maps can be seen in Figure 2. The analyses were based on difference scores normalized to eliminate amplitude differences using the mean maximum-minimum method (McCarthy and Wood, 1985). The ANOVA employed the same factors as the amplitude analysis although condition here refers to recent and remote old-new effects. The topographical analysis at the 300–500 ms window found no significant main effects of or interactions with condition (all *ps* > 0.05). The same comparison at the 600–800 ms period found a significant condition × site interaction (*F*_(1.22,18.23)_ = 4.39, *p* = 0.044) driven by the inverted polarity of the remote old-new effects at parietal sites. Such differences may be taken to indicate that recent and remote effects at this time window reflect the operation of partially distinct neuronal circuits involved in recent and remote recognition (Wilding, 2006).

The ERP findings have revealed important differences and similarities in the electrophysiological correlates of recent and remote recognition. Both elicit the FN400 which remains unaffected by the manipulation of delay. By contrast, recent but not remote recognition is associated with the LPE. Considering the hypothesized functional roles of the FN400 and the LPE as electrophysiological correlates of familiarity and recollection respectively, these results suggest a lack of episodic information within remote hits and a reliance on the overall sense of familiarity triggered by the pictorial cues.

However, before discussing the findings in more detail, it is important to confirm these assumptions. In particular, although there is strong evidence that the LPE is a correlate of recollection, the functional significance of the FN400 has been debated and it is not universally accepted as a correlate of familiarity (see for example, Paller et al., 2007). It is, therefore, important to establish a correspondence between behavioral and electrophysiological evidence. A previous behavioral study (Gardiner and Java, 1991), which explored recollection and familiarity at similar retention intervals, reported an accelerated loss of episodic details, consistent with our interpretation of the ERP data. In the following experiment, we sought to confirm the pattern of forgetting for recollection and familiarity under experimental conditions that were as close as possible to those in Experiment 1. This would allow stronger conclusions to be drawn regarding the functional significance of the ERP results.

## Experiment 2

### Introduction

The purpose of Experiment 2 was to identify with greater accuracy the contribution of recollection and familiarity processes at recent and remote recognition. The expectation from the results of Experiment 1 and previous findings (Gardiner and Java, 1991; Dudukovic and Knowlton, 2006; Roberts et al., 2013) was that whereas recent picture recognition would rely primarily on recollection, remote recognition would be based mainly on familiarity.

Experiment 2 was a behavioral-only study which required participants to provide an additional recollection or familiar judgement for items identified as previously studied. Detailed instructions (following Gardiner and Java, 1991) were provided to allow participants to differentiate between the two judgements. The only difference is that we used the terms “Recollect” and “Familiar” instead of “Remember” and “Know”, respectively (see Montaldi et al., 2006, for a similar approach). Although the latter terms have been widely used, they could be misinterpreted by participants. The word “remember” in everyday use could denote confidence in one’s memory judgements (irrespective of the presence of recollection) whereas “know”—as per Tulving (1985) original intention, is better suited to testing existing knowledge (i.e., semantic memory) than conveying a sense of familiarity due to an item’s recent exposure.

### Materials and Methods

#### Participants

Fourteen students (nine female; Age range: 21–29 years; Mean age = 24 years) from the University of Manchester participated in this experiment in return for a fixed £10 payment. All participants had normal or corrected to normal vision. The study was approved by the Ethics Committee of the School of Psychological Sciences, University of Manchester. Informed consent was obtained from all participants.

#### Materials

The materials were the same as in Experiment 1.

#### Procedure

The average period between the remote study session and test was 28 days (*SD* = 2.65; range = 24–32) whereas for items studied in the second session the average delay between study and test was 7 min (*SD* = 3.13; range = 2–14 min). At test, each trial commenced with a centrally presented fixation “O” which remained on screen for 300 ms and was replaced by a 150 ms blank interval. Each test item was shown for 600 ms and was then replaced by a blank screen. A centrally displayed fixation cross was presented concurrently on the screen and remained present until a response was made (as in Experiment 1). Participants were instructed to first decide whether an item was presented before (in either study phases) or whether it was new. If a new response was made there was a 1500 ms delay before the start of the next trial. If an old judgement was made, the words “recollect or familiar” were displayed at the center of the screen. At this point, participants had to decide whether their memory for the item was based on recollection and, therefore, accompanied by retrieval of specific episodic details (perceptual, conceptual associations etc.) or by a sense of familiarity in the absence of any additional episodic information. Different sets of response keys were employed for old/new and recollection/familiarity judgements. The allocation of response keys to different judgements was counterbalanced across participants. The test was subject-paced.

## Results and Discussion

The results of Experiment 2 are presented in Table 1. Examination of overall recognition revealed that as in Experiment 1, participants showed significant forgetting, retrieving fewer remote than recent items (*t*_(13)_ = −10.88, *p* < 0.001). As in Experiment 1, remote memory performance was better than chance with the remote hit rate significantly higher than the false alarm rate (*t*_(13)_ = 6.96, *p* < 0.001). Although the pattern of recognition performance in the two experiments was similar, a direct comparison showed that remote hits and false alarms were significantly lower in Experiment 2 than in Experiment 1 (remote hits: *t*_(28)_ = 2.25, *p* = 0.033; false alarms: *t*_(28)_ = 2.08, *p* = 0.047). On the other hand, recent hits did not differ between the two experiments. This is consistent with the adoption of a more conservative response criterion in Experiment 2, which would have resulted in weaker memories such as remote hits and false alarms being rejected. A comparison of response bias scores (*c* scores; Snodgrass and Corwin, 1988) found significant differences between the two experiments for remote but not recent memories (Remote memory: Experiment 1–*M* = 0.42 *SD* = 0.40 and Experiment 2–*M* = 0.83, *SD* = 0.53, *t*_(28)_ = −2.39, *p* = 0.024; Recent memory: Experiment 1–*M* = −0.20, *SD* = 0.29 and Experiment 2–*M* = −0.02, *SD* = 0.37, *t*_(28)_ = −1.46, *p* = 0.156). It is possible that the more detailed memory judgements required in Experiment 2, encouraged a more cautious approach.

The most important aspect of Experiment 2 is the breakdown of recent and remote performance in terms of the underlying memory processes. Inspection of recollection and familiar scores in Table 1 shows a disproportionate reduction in episodic retrieval. A 2 × 2, repeated measures ANOVA employing the factors of delay (remote vs. recent) and judgement (recollect vs. familiar) revealed, as expected, a main effect of delay (*F*_(1,13)_ = 117.55, *p* < 0.001) with more recent than remote hits. There was no main effect of judgement (*F*_(1,13)_ = 0.28, *p* = 0.60) but the delay by judgement interaction was significant (*F*_(1,13)_ = 22.42, *p* < 0.001). Recollection was reduced at the remote relative to the recent delay (*t*_(13)_ = 9.90, *p* < 0.001) whereas familiar judgments did not differ (*t*_(13)_ = 0.91, *p* = 0.38).

The marked reduction in recollection together with the preservation of familiar responses between the immediate and the four-week delay are in line with Gardiner and Java (1991). Although remote recollection in their study was much greater than in our study (0.19 vs. 0.06, respectively), remote familiar hit rates were very similar (0.21 in Gardiner and Java vs. 0.24 in the present study). A similar pattern of results was also obtained by Roberts et al. (2013) and Dudukovic and Knowlton (2006). In both these studies, comparisons between recognition following an immediate and a one-week retention interval revealed that, whereas Remember responses decreased, Know responses actually increased. This latter finding likely reflects a process whereby items that can be recollected immediately after study lose any associated episodic details at longer delays (Dudukovic and Knowlton, 2006).

The behavioral data from Experiment 2 have clear implications regarding the functional significance of ERPs in Experiment 1. Whereas recent memory ERPs contain both recollection and familiar hits, remote memory ERPs memory consist mainly of familiarity driven responses. An inspection of individual scores showed that 4 of the 14 participants did not report any remote recollections. Assuming equivalence across experiments, such a low percentage of remote recollections would have had minimal impact on the grand average ERP scores and could help explain the absence of the LPE for remote recognition in Experiment 1.

## General Discussion

Taken together the electrophysiological and behavioral results show a clear dissociation in the processes underlying recent and remote recognition. The first important finding to emerge from the present study is that the FN400 is not a short-lived effect but can be found at delays of at least four weeks. This extends previous findings who report robust effects at shorter testing intervals (Curran and Friedman, 2004; Wolk et al., 2006; Jaeger et al., 2009; Roberts et al., 2013). In terms of its functional significance, the ERP findings are also in agreement with the notion that the FN400 is a correlate of familiarity (e.g., Rugg and Curran, 2007). Remote recognition in Experiment 2 was driven almost exclusively by item familiarity and in Experiment 1 ERPs produced the FN400 but not the LPE. The second finding of interest is that neither the topography nor the amplitude of the FN400 showed sensitivity to memories’ age. This lack of modulation (in agreement with the behavioral data of Experiment 2, which showed similar rates of familiarity responses for recent and remote memories) suggests that the effect remains invariable at much longer delays than previously thought (Curran and Friedman, 2004; Wolk et al., 2006; Jaeger et al., 2009; Roberts et al., 2013).

To the extent that FN400 indexes familiarity, the implications of these findings are two-fold. First, they suggest that the neural generators of familiarity, as much as these can be determined by topographical analyses (see Urbach and Kutas, 2002; Wilding, 2006, for a detailed discussion of this issue) do not change as a function of time, for at least up to the four-week period examined in the present study. The second implication is that recent and remote familiar responses do not differ in terms of their underlying memory strength. This second point stems from evidence linking the amplitude of the FN400 to memory strength with weaker memories producing an attenuated effect (Azimian-Faridani and Wilding, 2006; Woodruff et al., 2006; Yu and Rugg, 2010).

This second point is, however, in doubt. There is evidence that recent and remote memories differ in memory strength. In a supplementary behavioral study and using a three-point memory strength judgement, Roberts et al. (2013) found that after one week, Know responses were associated with significantly weaker memories than immediate Know responses. At longer intervals such as the four week delay used in the present study, one would expect that strength differences between immediate and remote recognition would at the very least remain stable or, more likely, increase. The absence of modulation in the FN400 in Experiment 1 goes against these findings. It is possible, however, that the net familiarity difference between recent and remote recognition was not of sufficient magnitude to become evident in the electrophysiological data. Furthermore, some studies have also failed to find a link between memory strength and FN400 amplitude (Curran, 2004; De Chastelaine et al., 2009).

As mentioned earlier an alternative functional interpretation of the FN400 is that it represents an electrophysiological correlate of conceptual priming (e.g., Paller et al., 2007). Regarding retention effects, there has been relatively little research into the longevity of conceptual priming and its neural correlates. Robust priming at delays of up to three weeks has been found in a word-stem completion task (Goshen-Gottstein and Kempinsky, 2001). An fMRI study (Meister et al., 2007), using a semantic decision task found reliable priming effects at three days which correlated with reduced activity in the left inferior and middle frontal gyrus, areas previously linked to conceptual priming (e.g., Wig et al., 2005). A more recent study which compared directly the hemodynamic correlates of immediate and delayed (at 2 days) object naming priming, found greater suppression for long-term priming in the middle temporal gyrus, an area related to semantic lexical processing (Heath et al., 2012). In the absence (to our knowledge) of any ERP studies examining long-term conceptual priming, both its electrophysiological correlates and its relation to immediate priming effects remain unknown. Irrespective of the effect’s association with either familiarity or priming, which given its importance is likely to motivate further research, the absence of a modulation suggests that the FN400 reflects processes that are delay-invariant at least up to the period examined in the present study.

Regarding the LPE, the present results are in agreement and extend the findings of Roberts et al. (2013). Both studies demonstrate significant effects of retention interval, which underscore the close dependency between LPE magnitude and levels of recollection in memory performance. In the Roberts et al. study remote recollection produced robust LPE, which was nevertheless reduced relative to recent recollections. In the present study the LPE for remote recognition was absent as memory at the one month delay was likely to involve a very small number of recollected memories as shown in Experiment 2. Variations in LPE magnitude as a function of amount of recollected information (Wilding, 2000; Vilberg et al., 2006) suggest that the progressive attenuation of the LPE is a direct consequence of the loss of episodic details with the passage of time. Although the exact time course of this attenuation has yet to be determined, the high correlation with behavioral measures of recollection (Gardiner and Java, 1991) would predict a rapid initial reduction within the first few days after encoding followed by a more gradual decrease.

In terms of the neural generators of remote recognition, the only study to our knowledge which has looked at the hemodynamic correlates of remote recognition within a time period similar to the present study is by Takashima et al. (2006). In an fMRI experiment they looked at activity both immediately and at a 90 day delay. The hippocampus, a structure known to support recollection (e.g., Aggleton and Brown, 1999; Montaldi and Mayes, 2010) showed a reduction in activity with delay whereas a region in the ventral medial prefrontal cortex showed a concomitant increase. Given its deep-seated location and closed field formation, it is unlikely that the hippocampus contributes directly to scalp recorded EEG (Fernández et al., 1999). Indirectly, however, as part of a wider recollection network involving cortical sites in areas such as the parietal cortex (Woodruff et al., 2006; Vilberg and Rugg, 2007), the functional integrity of the hippocampus is essential to the emergence of the LPE (Düzel et al., 2001; Addante et al., 2012a). Evidence from a recent combined ERP/fMRI recognition memory also showed a correlation between LPE amplitude and activity in the right hippocampus among other areas (Hoppstädter et al., 2015). The fact that in the present study we could not detect an LPE for remote recognition is therefore consistent with Takashima et al’s findings. Familiarity on the other hand has been linked to areas including the dorsolateral prefrontal, perirhinal, parahippocampal and temporopolar cortex and the intraparietal sulcus (Montaldi et al., 2006; Bowles et al., 2007; Aly et al., 2011; see Ranganath and Ritchey, 2012 for a review) whereas FN400 amplitude has been found to correlate with activity in right ventrolateral prefrontal, right inferior parietal and left medial occipital cortex (Hoppstädter et al., 2015). Although Takashima et al. reported activity in a number of lateral prefrontal and parietal areas at the immediate recognition test, they did not report any change of activity in these areas or familiarity related areas with remote recognition. Therefore, to our knowledge, the hemodynamic correlates of familiarity memory at longer retention intervals remain unknown.

Another interesting yet unexpected aspect of the present results was that although remote hits did not produce an LPE, during the same period and at parietal electrodes, they elicited activity that was more negative going than correct rejections. The topography of the effect and its latency resemble the so-called late posterior negativity or LPN (for a review, see Johansson and Mecklinger, 2003). This is a heterogeneous effect thought to comprise at least two functionally and temporally distinct components: an early effect (600–1200 ms) associated with the search for episodic information and a late effect (1200–1900 ms) linked to the maintenance of a retrieved episodic memory (Herron, 2007). The onset of the effect in the present study at around 500–600 ms is close to the earlier of the two components. It is reasonable to assume that the long retention interval would have affected both the quality and strength of the underlying remote representations, which in turn would have reduced retrieval fluency and similar to a source retrieval task would have forced participants to engage in more effortful memory search. An alternative interpretation^3^ comes from a recent study (Addante et al., 2012b) in which items that were less confidently recognized but nevertheless accompanied by correct source retrieval elicited, at around 600 ms, activity that was more negative going compared to correct rejections. Addante et al. (2012b) suggested that this parietal negativity reflected contextual familiarity, a form of familiarity, which according to a recent theoretical model (Montaldi and Mayes, 2010) supports the activation of contextual features associated with a recognized item in the absence of recollection. There are however problems with both interpretations. Although present with item recognition, LPN is mostly seen in tasks that require source retrieval (Donaldson and Rugg, 1998; Friedman et al., 2005; Herron, 2007) and both the LPN and Addante et al. (2012b) contextual familiarity negativity tend to be longer lasting than the effect in the present study, which as can be seen in Figure 1 had a much shorter duration (see also Leynes and Zish, 2012; Tibon et al., 2014; for similar findings). Also, the contextual familiarity negativity effect had a frontal distribution during the 600–800 ms window unlike the parietal focus in the present study. Behaviorally, the contextual familiarity in Addante et al. (2012b) was presumed to underlie and support correct source retrieval for less confident recognized items. If remote recognition was accompanied by contextual familiarity in the present study, it is possible that the activation of contextual features (such as time or place) could have been sufficient to support “Recollect” judgments for remote memories (in a manner similar to source retrieval). But, the very low recollection rates in Experiment 2 suggest that this was not the case. As this brief discussion demonstrates, more research is needed to identify the functional significance of the observed parietal negativity and the way in which the processes it represents are linked to remote recognition.

## Summary and Conclusions

The present study showed that the electrophysiological correlates of remote recognition differ markedly from those of recent recognition. Remote recognition ERPs were characterized by the presence of FN400 and the absence of the LPE. The purported functional roles of these two effects as the electrophysiological correlates of familiarity and recollection respectively, are in agreement with behavioral findings which show a marked loss of episodic information in the first few weeks after learning. Future studies would be needed to shed more light into the relationship between the FN400 and retention interval and by extension between the FN400 and memory strength.

## Author Contributions

DT was responsible for the design of the study. DT and NW were responsible for data acquisition. DT and WED were responsible for data analysis. All authors were involved in manuscript writing and approved the final version of this manuscript.

## Conflict of Interest Statement

The authors declare that the research was conducted in the absence of any commercial or financial relationships that could be construed as a potential conflict of interest.

## Footnotes

^1^http://wiki.cnbc.cmu.edu/Objects

^2^http://www.easycap.de

^3^We would like to thank one of our reviewers for suggesting this possibility.
